# Supplementary material for: Interspecific Host Variation and Biotic Interactions Drive Pathogen Community Assembly in Chinese Bumblebees
Source: Insects. 2023 Nov 17;14(11):887. doi: 10.3390/insects14110887 (PMC10672019; doi:10.3390/insects14110887)
Supplement: Supplementary file 1 [file insects-14-00887-s001.zip › Chen_et_al_supporting_Table S1.pdf]

Table S1. The information of sample sites and number of bumblebees collected in this study

**Province**	**County**	**Site**	**Altitude**	**Species**	**Number**
Hebei	Longhua	Longhua	553	*B. patagiatus*	30
				B. ignitus	30
Hebei	Xinglong	Wulingshan	905	*B. patagiatus*	30
				B. ignitus	24
Shanxi	Fangshan	Beiwudang	1372	*B. patagiatus*	15
Shanxi	Jiaochen	Pangquangou	1482	*B. pyrosoma*	30
Yunnan	Kaiyuan	Dazhuang	2467	*B. friseanus*	30
Yunnan	Mengzi	Lengquan	1583	*B. breviceps*	30
Jiangsu	Yixing	Hufu	66	*B. trifasciatus*	30
Jiangxi	Nanchang	Wanli_Meiling	186	*B. trifasciatus*	30
Shannxi	Zhashui	Yingpan	1116	*B. longipes*	14
Shannxi	Yangxian	Huayang	2289	*B. trifasciatus*	30
Zhejiang	Hangzhou	Chunan_Zhaitang	131	*B. braccatus*	28
Xinjiang	Hejing	Dukugonglu	2820	*B. lucorum*	18
				B. keriensis	29
Xinjiang	Mulei	Shuimogou	1608	*B. cryptarum*	22
				B. turkestanicus	30
				B. cullumanus	15
				B. subterraneus	29
				B. soroeensis	5
				B. armeniacus	14
Qinghai	Guide	Garang	2777	*B. lantschouensis*	30
Gansu	Huixian	Lichuan	1520	*B. longipes*	9
Gansu	Diebu	Zhagana	3277	*B. convexus*	16
				B. rufofasciatus	30
Gansu	Tianzhu	Shimen	2612	*B. waltoni*	20
Gansu	Zhuoni	Daogao	2846	*B. lepidus*	30
				B. pyrosoma	30
Gansu	Kangle	Huguan	1978	*B. pyrosoma*	30
Sichuan	Nuoergai	Tangke	3500	*B. friseanus*	16
				B. keriensis	29
				B. sushkinis s.l.	18
**Total numbers**					**771**
