# Supplementary material for: Interspecific Host Variation and Biotic Interactions Drive Pathogen Community Assembly in Chinese Bumblebees
Source: Insects. 2023 Nov 17;14(11):887. doi: 10.3390/insects14110887 (PMC10672019; doi:10.3390/insects14110887)
Supplement: Supplementary file 1 [file insects-14-00887-s001.zip › Chen_et_al_supporting_Table S2.pdf]

Table S2. Primers used for PCR amplification

Target DNA	Primer	Sequence(5'-3')	Reference
Apicystis bombi	NeoF	CCAGCATGGAATAACATGTAAGG	*Meeus et al.,*2010
	NeoR	GACAGCTTCCAATCTCTAGTCG	
Nosema bombi	Nbombi-SSU-Jf1	CCATGCATGTTTTTGAAGATTATTAT	*Klee et al.,*2006
	Nbombi-SSU-Jr1	CATATATTTTTAAAATATGAAACAATAA	
Crithidia bombi	CB-Cytb2-F	GT(A/T)TT(G/A)TTTTT(G/A)TG(G/A)GATT TTG	*Schmid-Hempel et al.,*2010
	CB-Cytb2-B	CATAAACG(T/C)TCACAATAAAATGC	
Locustacarus buchneri	Lb64F	GGGAAGGTACAGGAACTGGATGAACT	*Arismendi et al.,*2016
	Lb417R	GAATGTAGACCTCTGGGTGTCCGAA	
Nosema apis	N. apis F	CCATTGCCGGATAAGAGAGT	*Chen et al.,*2008
	N. apis R	CACGCATTGCTGCATCATTGAC	
Nosema ceranae	N. ceranae F	CGGATAAAAGAGTCCGTTACC	*Chen et al.,*2008
	N. ceranae R	TGAGCAGGGTTCTAGGGAT	
Black queen cell virus	BQCV1	TGGTCAGCTCCCACTACCTTAAAC	*Benjeddou et al.,*2001
	BQCV2	GCAACAAGAAGAAACGTAAACCAC	
Deformed wing virus	DWV-T3-fwd	GTCGTGCAGCTCGATAGGAT	*Tentcheva et al.,2004*
	DWV-T3-rev	TTTGCAAGATGCTGTATGTGG	
Acute bee paralysis virus	ABPVYZF	GAGATGACTGGGGAGGAGTGATA	This study
	ABPVYZR	GTCCTGAAAGTAAAGGCTTAGGTTC	
Israeli acute paralysis virus	IAPVF	AGACACCAATCACGGACCTCAC	*Hou et al.,*2014
	IAPVR	AGATTTGTCTGTCTCCCAGTGCACAT	
Chronic bee paralysis virus	CBPV1-1	TCAGACACCGAATCTGATTATTG	*Blanchard et al.,*2008
	CBPV1-2	ACTACTAGAAACTCGTCGCTTCG	
Sacbrood virus	SBV-qF3164	TTGGAACTACGCATTCTCTG	*Locke et al.,*2012
	SBV-qB3461	GCTCTAACCTCGCATCAAC	

Arismendi, N., Bruna, A., Zapata, N. and Vargas, M. (2016) Molecular detection of the tracheal mite *Locustacarus buchneri* in native and non-native bumble bees in Chile. *Insectes Sociaux*, 63, 629-633.

Benjeddou, M., Leat, N., Allsopp, M. and Davison, S. (2001). Detection of acute bee paralysis virus and black queen cell virus from honeybees by reverse transcriptase PCR. *Applied and Environmental Microbiology*, 67, 2384–2387.

Blanchard, P., Olivier, V., Iscache, A.L., Celle, O., Schurr, F., Lallemand, P. *et al*. (2008) Improvement of RT-PCR detection of chronic bee paralysis virus (CBPV) required by the description of genomic variability in French CBPV isolates. *Journal of Invertebrate Pathology*, 97, 182–185.

Chen, Y.P., Evans, J.D., Smith, I.B. and Pettis, J.S. (2008) *Nosema ceranae* is a long-present and wide-spread microsporidian infection of the European honey bee (*Apis mellifera*) in the United States. *Journal of Invertebrate Pathology*, 97, 186–188.

Hou, C.S., Rivkin, H., Slabezki, Y. and Chejanovsky, N. (2014) Dynamics of the presence of Israeli acute paralysis virus in honey bee colonies with colony collapse disorder. *Viruses*, 6, 2012–2027.

Klee, J., Tay, W.T. and Paxton, R.J. (2006). Specific and sensitive detection of *Nosema bombi* (Microsporidia: Nosematidae) in bumble bees (*Bombus* spp.; Hymenoptera: Apidae) by PCR of partial rRNA gene sequences. *Journal of Invertebrate Pathology*, 91, 98–104.

Locke, B., Forsgren, E., Fries, I. and de Miranda, J.R. (2012) Acaricide treatment affects viral dynamics in *Varroa destructor*-infested honey bee colonies via both host physiology and mite control. *Applied & Environmental Microbiology*, 78, 227–235.

Meeus, I, de Graaf, D.C., Jans, K. and Smagghe, G. 2010. Multiplex PCR detection of slowly-evolving trypanosomatids and neogregarines in bumblebees using broad-range primers. *Journal of Applied Microbiology*, 109, 107–115.

Schmid-Hempel, R. and Tognazzo, M. (2010) Molecular divergence defines two distinct lineages of *Crithidia bombi* (Trypanosomatidae), parasites of bumblebees. *Journal of Eukaryotic Microbiology*, 57, 337–345.

Tentcheva, D., Gauthier, L., Jouve, S., Canabady-Rochelle, L. and Bergoin, M. (2004) Polymerase chain reaction detection of deformed wing virus (DWV) in *Apis mellifera* and *Varroa destructor*. *Apidologie*, 35, 431–439.
